# Executive Dysfunctions and Event-Related Brain Potentials in Patients with Amyotrophic Lateral Sclerosis

**DOI:** 10.3389/fnagi.2015.00225

**Published:** 2015-12-18

**Authors:** Caroline Seer, Stefanie Fürkötter, Maj-Britt Vogts, Florian Lange, Susanne Abdulla, Reinhard Dengler, Susanne Petri, Bruno Kopp

**Affiliations:** ^1^Department of Neurology, Hannover Medical SchoolHannover, Germany; ^2^Department of Neurology, Otto-von-Guericke University MagdeburgMagdeburg, Germany; ^3^Department of Neurology, German Center for Neurodegenerative DiseasesMagdeburg, Germany

**Keywords:** amyotrophic lateral sclerosis, ERP, LRP, executive functions, attention

## Abstract

A growing body of evidence implies psychological disturbances in amyotrophic lateral sclerosis (ALS). Specifically, executive dysfunctions occur in up to 50% of ALS patients. The recently shown presence of cytoplasmic aggregates (TDP-43) in ALS patients and in patients with behavioral variants of frontotemporal dementia suggests that these two disease entities form the extremes of a spectrum. The present study aimed at investigating behavioral and electrophysiological indices of conflict processing in patients with ALS. A non-verbal variant of the flanker task demanded two-choice responses to target stimuli that were surrounded by flanker stimuli which either primed the correct response or the alternative response (the latter case representing the conflict situation). Behavioral performance, event-related potentials (ERP), and lateralized readiness potentials (LRP) were analyzed in 21 ALS patients and 20 controls. In addition, relations between these measures and executive dysfunctions were examined. ALS patients performed the flanker task normally, indicating preserved conflict processing. In similar vein, ERP and LRP indices of conflict processing did not differ between groups. However, ALS patients showed enhanced posterior negative ERP waveform deflections, possibly indicating increased modulation of visual processing by frontoparietal networks in ALS. We also found that the presence of executive dysfunctions was associated with more error-prone behavior and enhanced LRP amplitudes in ALS patients, pointing to a prefrontal pathogenesis of executive dysfunctions and to a potential link between prefrontal and motor cortical functional dysregulation in ALS, respectively.

## Introduction

Amyotrophic lateral sclerosis (ALS) is a devastating neurodegenerative disease characterized by combined degeneration of upper and lower motor neurons. The resulting progressive paralysis involving loss of bulbar and limb muscle function eventually causes death due to respiratory failure within an average of 3 years after symptom onset (Wijesekera and Leigh, 2009). The incidence of ALS in Europe is 2.16 per 100,000 persons (Logroscino et al., 2010), its etiology still remains largely unknown (Strong and Rosenfeld, 2003; Kiernan et al., 2011; Turner et al., 2013).

While ALS has traditionally been thought to be restricted to the motor nervous system, it is now increasingly acknowledged as a multiple system disease, also affecting non-motor areas of the cortex (Kew et al., 1993a; Abrahams et al., 1996; Geser et al., 2008; Wijesekera and Leigh, 2009; Kiernan et al., 2011; Sarro et al., 2011; Tsermentseli et al., 2012; Agosta et al., 2013; Pettit et al., 2013; Turner et al., 2013; Swinnen and Robberecht, 2014; Turner and Swash, 2015). About 30–50% of the ALS patients have been reported to develop mild cognitive and behavioral disturbances, with 5–15% fulfilling the criteria for frontotemporal dementia (FTD; Neary et al., 1998; Lomen-Hoerth et al., 2003; Ringholz et al., 2005; Elamin et al., 2011; Phukan et al., 2012). Behavioral changes accompanying FTD in ALS may involve apathy, personality change, poor insight, and disinhibition, among others (Grossman et al., 2007; Gibbons et al., 2008; Witgert et al., 2010; Abrahams, 2011; Lillo et al., 2011). Cytoplasmic aggregates of the transactive response DNA binding protein (TDP)-43 have been identified in ubiquitinated neuronal inclusion in post mortem brain tissue in ALS patients and in a subgroup of FTD patients, indicating that these disease entities belong to a common disease spectrum (Arai et al., 2006; Neumann et al., 2006; Geser et al., 2008). The overlap of ALS and FTD is further backed up by recent findings in genetics, in particular by the detection of mutations in the TARDP-gene coding for TDP-43 and, more importantly, of hexanucleotide expansions in the C9ORF72 gene as causal for ALS and FTD (DeJesus-Hernandez et al., 2011; Renton et al., 2011; Al-Chalabi et al., 2012; Robberecht and Philips, 2013). Moreover, alterations common to ALS and FTD have been revealed by functional imaging studies. Specifically, neuronal degeneration in prefrontal regions, including dorsolateral prefrontal cortex (dlPFC), and anterior cingulate (ACC), has been linked to some of the cognitive changes of ALS patients (Abrahams et al., 1996, 2005a; Tsermentseli et al., 2012; Pettit et al., 2013). Importantly, not only comorbid FTD (Olney et al., 2005; Elamin et al., 2011) but also executive dysfunction in non-demented ALS patients has been found to be associated with shorter survival times (Elamin et al., 2011).

Non-motor involvement in patients with ALS has been commonly described to manifest in executive dysfunctions (Neary et al., 2000; Phukan et al., 2007; Raaphorst et al., 2010; Goldstein and Abrahams, 2013). Specifically, patients have been found to be impaired in selective attention (Chari et al., 1996; Massman et al., 1996; Abrahams et al., 1997; Pinkhardt et al., 2008; Christidi et al., 2012), verbal fluency (Ludolph et al., 1992; Kew et al., 1993a; Lomen-Hoerth et al., 2003; Massman et al., 1996; Abe et al., 1997; Frank et al., 1997; Rakowicz and Hodges, 1998; Abrahams et al., 2000, 2004, 2005b; Phukan et al., 2012), and cognitive flexibility (Neary et al., 1990; Massman et al., 1996; Abrahams et al., 1997; Moretti et al., 2002; Lange et al., 2015; but see Ludolph et al., 1992; Kew et al., 1993a).

Although non-motor symptoms seem to be common in patients with ALS (Strong et al., 2009), mild cognitive change is difficult to assess in ALS patients due to potential motor and speech deficiencies (Goldstein and Abrahams, 2013). The event-related potentials (ERP) technique (Luck, 2005; Raggi et al., 2010) provides an excellent tool to assess cognitive (dys)functions in patients with ALS under minimal motor demands and with high temporal resolution (Neumann and Kotchoubey, 2004; Raggi et al., 2008, 2010; Goldstein and Abrahams, 2013; Lange et al., 2015). ERP waveforms are usually subdivided into early ERP components whose characteristics are associated with physical features of the eliciting stimuli (“exogenous”) and late ERP components whose characteristics are associated with cognitive (“endogenous”) processing of the stimuli, such as the negative-going fronto-centrally distributed N2 component (Folstein and van Petten, 2008) and the positive-going parietally distributed P3 component (Polich, 2007). Previous ERP studies revealed that ALS patients show altered endogenous ERP waveforms (Raggi et al., 2010). Specifically, the N2 and P3 latencies were prolonged in ALS patients in perceptual discrimination tasks (Gil et al., 1995; Paulus et al., 2002; Amato et al., 2013). Further, P3 amplitudes were decreased in ALS patients in perceptual discrimination and visual search paradigms (Münte et al., 1999; Hanagasi et al., 2002; Raggi et al., 2008). Contrarily, irrelevant distractor stimuli in an auditory selective attention task were reported to elicit enhanced P3 amplitudes and shortened P3 latencies in ALS patients (Pinkhardt et al., 2008). Taken together, these ERP abnormalities have been interpreted as indices for disturbances in cortical processing for selective attention and executive processing in patients with ALS (Raggi et al., 2010).

Conflict processing represents an important dimension of executive functions (Botvinick et al., 2001). Previous research on conflict processing in ALS mainly focused on the Stroop task, a color-word conflict task, with inconsistent behavioral findings (Ludolph et al., 1992; Kew et al., 1993a; Abrahams et al., 1997; Frank et al., 1997; Thorns et al., 2010; Goldstein et al., 2011; Phukan et al., 2012; Zalonis et al., 2012). The present study aimed to contribute to the literature on executive dysfunctions in ALS by examining electrophysiological correlates of conflict processing. Since language impairments are highly prevalent in ALS patients (Abrahams, 2013; Taylor et al., 2013), we used a non-verbal conflict task for this purpose to avoid potential language confounds (Kopp et al., 1996).

**Figure 1:**
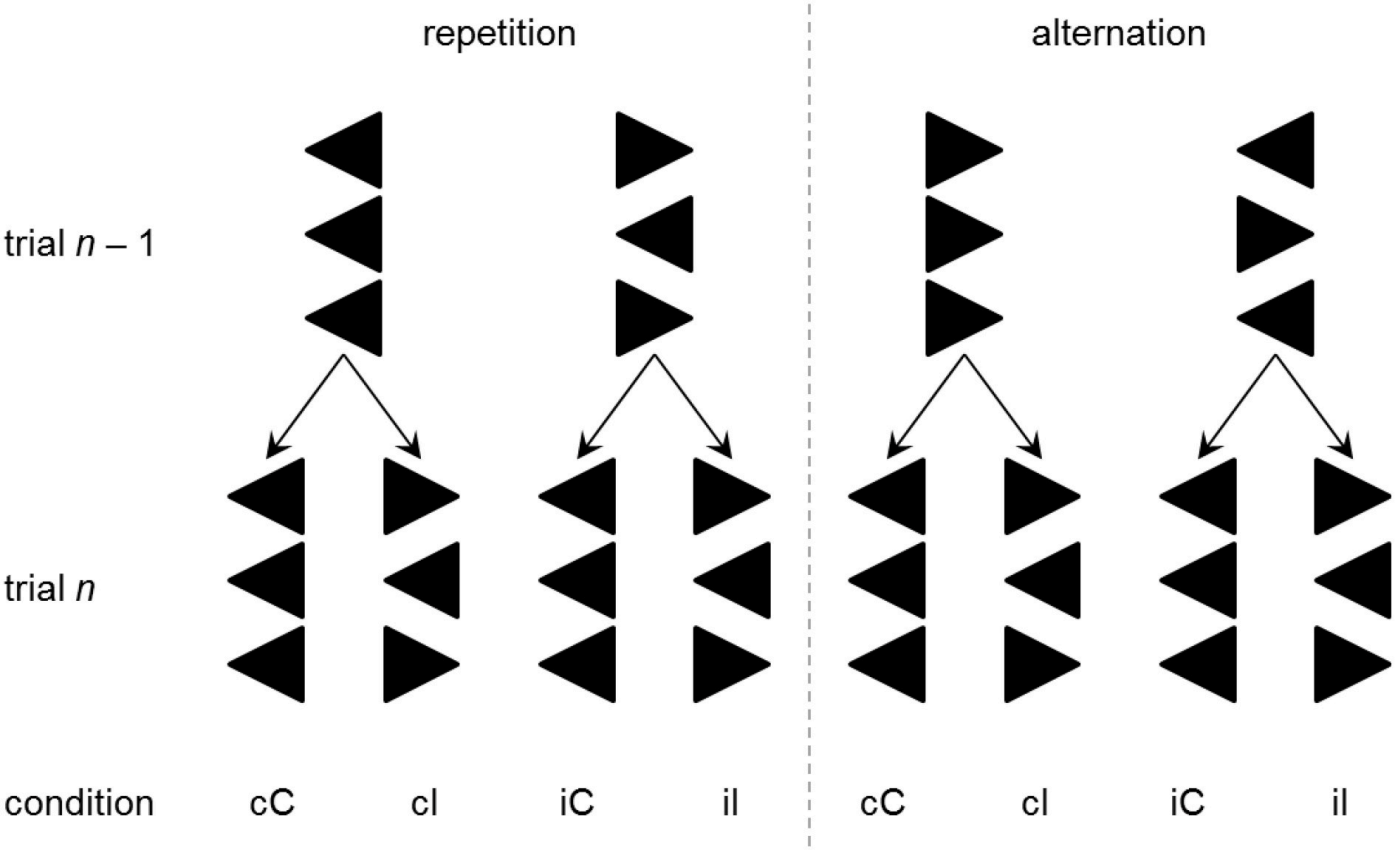
**Four possible congruency sequences (*cC*, *cI*, *iC*, and *iI*) are obtained by factorial combination of previous trial (trial *n*–1) congruency (congruent vs. incongruent) and current trial (trial *n*) congruency**. Depending on the correct response hand (left vs. right), this constellation will require either a repetition (left panel) or alternation (right panel) of the motor response. Combination of previous congruency, current congruency, response sequence, and response hand yields 16 different stimulus arrays. Only eight arrays are displayed here, as the remaining arrays are mirror images.

The non-verbal variant of the Eriksen flanker task (Eriksen and Eriksen, 1974) requires the subject to respond to a central target stimulus (e.g., “>”) that is flanked symmetrically by either congruent (“>”) or incongruent (“ < ”) distractor stimuli (see Rustamov et al., 2013 for discussion). Assessment of response times (RT) and error rates (ER) allows estimating individual information processing abilities, with shorter RT and lower ER indicating more efficient processing. Typically, RT and ER are smaller for congruent situations (e.g., “> > >”) than they are for incongruent situations (e.g., “> < >”). This *congruency effect* (CE) is usually understood as an indicator of conflict processing. In addition, it has been observed that RTs and ERs are not only influenced by the congruency of target and flanker stimuli on any trial, but they are also contextually modulated by the congruency sequence, i.e., by the interaction between the congruency on the current trial and the congruency on the previous trial. Specifically, RT and ER benefits are typically found when a congruent trial was preceded by another congruent trial (this case is labeled *cC* trial throughout this paper) rather than by an incongruent trial (*iC*), and when an incongruent trial was preceded by another incongruent trial (*iI*) rather than by a congruent trial (*cI*). In the flanker task, this *congruency sequence effect* (CSE; Gratton et al., 1992; Egner, 2007) has been found to occur specifically when the response can be repeated across two successive trials, but to be completely absent when responses alternate across two successive trials (Rustamov et al., 2013). Figure 1 depicts the trial sequences of interest (*cC, iC, iI, cI*) separately for response repetition and response alternation trials. Inspection of Figure 1 reveals that *cC* and *iI* trials enable repetition priming (via exact stimulus repetitions) in response repetition, but not in response alternation trials. The specificity of the CSE for response repetition trials suggests that priming plays an important role in contextual modulation of conflict processing as assessed by the flanker task (Mayr et al., 2003). Until now, no study investigated conflict processing and its contextual modulation in ALS by means of the flanker task. However, Luks et al. (2010) found the CE in error rates (i.e., more errors in incongruent trials than in congruent trials) on the flanker task to be related to atrophy of the dlPFC and ACC—i.e., two prefrontal regions that have been related to the presence of cognitive impairments in patients with ALS (Abrahams et al., 1996; Goldstein et al., 2011; Tsermentseli et al., 2012; Pettit et al., 2013).

The ERP technique offers a fine-grained analysis of the cognitive processes as they occur in rapid succession during information processing. To begin with, posterior negative ERP waveform deflections indicate modulation of visual processing by frontoparietal attention networks (Ptak, 2012; Rustamov et al., 2014; Vossel et al., 2014). For example, selective attention to relevant stimulus features is typically associated with the appearance of a *selection negativity* (SN) at posterior electrode sites. The SN is a negative ERP waveform deflection that has its onset at around 160 ms post-stimulus and persists for approximately 200 ms and is thought to indicate prioritized processing of attended stimulus features (Hillyard and Anllo-Vento, 1998; Hillyard et al., 1998; Kopp et al., 2007; Kopp and Wessel, 2010). Compelling evidence that posterior negativities are subject to prefrontal modulation comes from a study of patients with unilateral focal prefrontal lesions. This study revealed that prefrontal damage reduced posterior negativities, putatively originating from neuronal activity in the extrastriate cortex of the lesioned hemisphere, during visual discrimination (Barceló et al., 2000).

The fronto-centrally distributed N2 component of the ERP is a negative-going potential that is typically observed around 250–300 ms post-stimulus. The N2 has been proposed to reflect conflict processing (Folstein and van Petten, 2008). Correspondingly, N2 amplitudes are typically enhanced on incongruent trials (i.e., stimuli are associated with a higher degree of conflict) of the Eriksen flanker task (Kopp et al., 1996; Yeung et al., 2004; Danielmeier et al., 2009). Likewise, the CSE has been associated with N2 modulations such that enhanced N2 amplitudes are observed on incongruent trials preceded by congruent trials (*cI*) than on incongruent trials preceded by incongruent trials (*iI*; Clayson and Larson, 2011a,b, 2012; Freitas et al., 2009; Forster et al., 2011; Larson et al., 2012; Rustamov et al., 2013). Interestingly, the individual differences with regard to the degree of these CSE-related N2 modulations correlate with cognitive performance in tests of attention, executive functions and verbal fluency (Clayson and Larson, 2012). In a recent study, Rustamov et al. (2013) found that N2 amplitudes were modulated by the congruency sequence in control participants, but the CSE-related N2 modulation was absent in patients with Parkinson's disease (PD).

The lateralized readiness potential (LRP; Coles, 1989; Eimer, 1998) has its neural generators in primary motor areas (Leuthold and Jentzsch, 2002) and it indexes the duration of the selection and of the preparation of appropriate motor responses. In the context of tasks requiring uni-manual responses, recordings from two electrodes over the primary motor areas can be used to calculate the LRP such that negative deflections indicate the activation and preparation of a uni-manual correct response. The stimulus-locked LRP (s-LRP) onset latency indicates the time interval between stimulus onset and the initiation of response preparation. Thus, s-LRP onset latencies provide a measure of the duration of pre-motor (perceptual and cognitive) processes (i.e., stimulus encoding plus response selection). In the context of the Eriksen flanker task, s-LRP onset latencies revealed that the preparation of the correct response started earlier on congruent trials than it did on incongruent trials (Gratton et al., 1988; Kopp et al., 1996).

Finally, LRP amplitudes provide measures of the functional dysregulation of cortical motor-generation processes. For example, normal aging is associated with an enhancement of LRP amplitudes (Yordanova et al., 2004; Roggeveen et al., 2007; Wild-Wall et al., 2008; Vallesi and Stuss, 2010; Cespón et al., 2013; Cid-Fernández et al., 2014). There is one published study which examined LRP amplitudes in ALS patients, and it reported diminished LRP amplitudes in a response inhibition paradigm (Thorns et al., 2010).

Here, we used a combination of behavioral and electrophysiological measures (ERP, LRP) obtained on the flanker task to examine conflict processing (CE) and its contextual modulation (CSE) in ALS patients. Several hypotheses about potentially distinct cognitive impairments are conceivable. Some of them are not mutually exclusive; nevertheless, they allow to study the dynamic interplay between selective attention, conflict processing and motor preparation. First, ALS patients may suffer from disturbed attentional modulation of visual processing (Chari et al., 1996; Massman et al., 1996; Abrahams et al., 1997; Pinkhardt et al., 2008; Christidi et al., 2012) that should express itself in altered posterior negativities. A corollary of such an attentional disturbance would be that flanker stimuli impose increased levels of conflict in ALS patients compared to controls, leading to enhanced behavioral (i.e., larger RT and ER differences between congruent and incongruent trials) and neural (i.e., disproportionally increased N2 amplitudes and prolonged s-LRP onset latencies on incongruent trials) indicators of conflict processing. Another possibility is that the cognitive disturbances in ALS are confined to impaired contextual modulation of conflict processing. This hypothesis leads to the prediction of altered behavioral and neural indicators of the CSE, as discussed above and as demonstrated in an earlier study from our group in patients with PD (Rustamov et al., 2013). Finally, ALS patients may show dysregulated cortical motor preparation processes, and this dysregulation should manifest itself in altered LRP amplitudes. We also assessed participants' performance on a number of neuropsychological tests in order to evaluate potential relationships between clinically manifest indicators of executive dysfunctions in ALS patients and behavioral and neural indices of conflict processing and its contextual modulation.

## Methods

### Participants

A cohort of 21 patients with ALS [15 males (71.4%); age: *M* = 58.90 years, *SD* = 9.62, range: 34–76; education years: *M* = 13.86 years, *SD* = 2.29, range: 10–19] was recruited between July 2013 and January 2014 from Hannover Medical School. Patients were included if they fulfilled the revised El Escorial criteria for clinically probable or definite ALS (Brooks et al., 2000). Eighteen patients had limb-onset disease, whereas three patients had bulbar-onset disease, and one patient fulfilled the criteria for FTD (Neary et al., 1998). Four patients were treated with nocturnal non-invasive ventilation (NIV) and none of the patients had a percutaneous endoscopic gastrostomy (PEG). A third-party rating (Abrahams et al., 2014) was administered to the patients' relatives or caregivers to assess potential behavioral alterations and psychotic symptoms. This information was available for 12 patients, and indicated that none of the patients showed behavioral disinhibition, whereas apathy and hyperorality/altered eating behavior were reported in two cases, and loss of sympathy/empathy and perseverative/stereotyped behavior were reported in three cases. One patient was reported to show psychotic tendencies. Criteria for exclusion involved other neurological diseases, any psychiatric disorder, and highly restricted pulmonary function. Furthermore, patients were excluded when they were too impaired to press a button of a keyboard due to their disease severity. Clinical status of ALS patients was investigated using the ALSFRS-EX (Abdulla et al., 2013; see Table 1), a 15-item self-report measure assessing functional impairments, which is an adapted and validated version of the well-established revised Amyotrophic Lateral Sclerosis Functional Rating Scale (ALSFRS-R; Cedarbaum et al., 1999). Respiratory function was quantified by the forced vital capacity (FVC), revealing an average FVC of 85.47 (*SD* = 14.62, range: 60–109). Additionally, daytime sleepiness was assessed using the Epworth Sleepiness Scale (ESS; Johns, 1991), revealing an average of 5.55 (*SD* = 3.17, range: 0–11). Twenty-one age-, gender-, and education-matched healthy (i.e., these participants were not diagnosed with ALS nor with any other neurological disease) controls (HC) were examined [age: *M* = 57.67 years, *SD* = 9.16, range 44–74 years; 15 males (71.4%); education years: *M* = 14.29 years, *SD* = 2.93, range = 10.5–20.5]. The HC group did not differ from ALS patients with regard to age, *t*_(40)_ = −0.43, *p* = 0.672, or education years, *t*_(40)_ = 0.53, *p* = 0.600. Control participants were either spouses or friends of the participants, or volunteers who were compensated for their participation with payment (30 €). One control participant had to be excluded from the analyses regarding the flanker task due to poor task comprehension. All participants were right-handed and had normal or corrected-to-normal vision and intact hearing. The study was reviewed and approved by the local ethics committee (Ethics Committee of Hannover Medical School: vote number 6269). All participants gave written informed consent in accordance with the Declaration of Helsinki.

**Table 1:** **Summary statistics on clinical characteristics and neuropsychological measures for ALS patients (ALS) and controls (HC) and ALS patients with (ALSef−) and without (ALSef+) executive dysfunctions according to the M-WCST EFC**.

	**max**.	**Mean (*SD*)**		***p*^a^**	**Mean (*SD*)**		***p*^b^**
		**HC**	**ALS**		**ALSef−**	**ALSef+**	
Duration since ALS onset (months)			43.29 (75.77)		42.80 (74.94)	47.30 (83.58)	0.901
ALSFRS-EX total	60		46.85 (7.18)		47.20 (5.61)	46.50 (8.77)	0.834
Bulbar subscore	16		13.65 (1.98)		13.50 (2.12)	13.80 (1.93)	0.745
Fine motor subscore	16		11.45 (2.91)		12.30 (3.02)	10.60 (2.67)	0.199
Gross motor subscore	16		10.85 (5.11)		10.00 (5.64)	11.70 (4.67)	0.472
Respiratory subscore	12		10.90 (1.74)		11.40 (1.26)	10.40 (2.07)	0.208
Progression rate			0.75 (0.54)		0.80 (0.55)	0.69 (0.55)	0.667
ECAS total	136	104.38 (11.58)	102.05 (13.12)	0.545	97.50 (15.02)	104.60 (9.08)	0.217
ECAS ALS-specific	100	75.48 (10.63)	74.10 (11.22)	0.684	68.70 (12.36)	77.90 (7.09)	0.056
Language	28	26.62 (1.94)	26.14 (2.06)	0.444	26.20 (2.15)	26.00 (2.16)	0.838
Fluency	24	11.52 (5.83)	10.19 (4.33)	0.405	8.40 (3.98)	11.00 (3.30)	0.129
Executive (EFS)	48	37.33 (4.50)	37.76 (7.18)	0.818	34.10 (8.66)	40.90 (3.25)	*0.039*
ECAS ALS-non-specific	36	28.86 (3.07)	27.95 (3.51)	0.380	28.80 (3.94)	26.70 (2.75)	0.184
Memory	24	17.24 (2.83)	16.48 (3.33)	0.428	17.40 (3.47)	15.10 (2.69)	0.115
Visuospatial	12	11.62 (0.74)	11.48 (0.68)	0.518	11.40 (0.70)	11.60 (0.70)	0.530
FAB	18	17.19 (1.25)	16.37 (2.19)	0.162	16.10 (2.33)	16.5 (2.20)	0.716
M-WCST EFC		106.81 (15.66)	96.15 (21.50)	0.079	77.70 (13.03)	114.60 (7.07)	<*0.001*
Categories	7	5.76 (1.55)	4.65 (2.16)	0.068	3.00 (1.89)	6.30 (0.48)	<*0.001*
Perseveration errors	47	1.33 (2.74)	3.40 (5.02)	0.115	6.60 (5.50)	0.20 (0.42)	*0.005*
MoCA	30	27.24 (2.68)	26.62 (3.06)	0.488	25.60 (3.13)	27.50 (2.95)	0.181

*ECAS, Edinburgh Cognitive and Behavioral ALS Screen; FAB, Frontal Assessment Battery; M-WCST, Modified Wisconsin Card Sorting Test; EFC, Executive Function Composite; MoCA, Montreal Cognitive Assessment*.

a *HC vs. ALS*.

b *ALSef− vs. ALSef+*.

A battery of neuropsychological tests was administered to the participants. The Edinburgh Cognitive and Behavioral ALS Screen (ECAS; Abrahams et al., 2014) was used to examine ALS-specific (executive functions, verbal fluency, language) as well as non-ALS-specific (memory, visuospatial functions) cognitive abilities. Frontal lobe functions were examined using the Frontal Assessment Battery (FAB; Dubois et al., 2000; Kopp et al., 2013). Participants were screened for mild cognitive impairment and dementia using the Montreal Cognitive Assessment (MoCA; Nasreddine et al., 2005).

**Figure 2:**
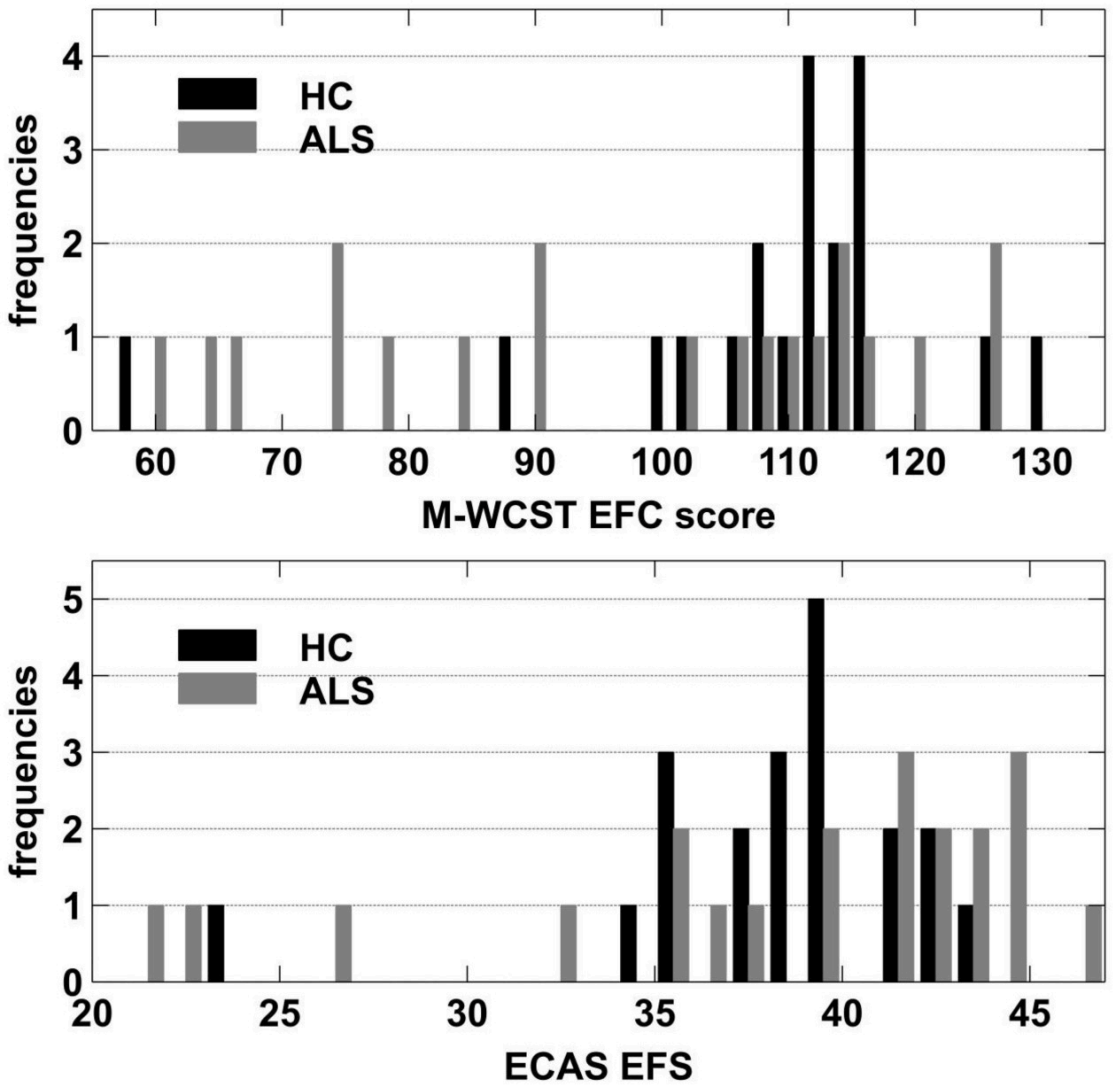
**Frequency histograms of M-WCST EFC scores (upper panel) and ECAS EFS (lower panel) separately for ALS patients and HC**.

To further assess potential effects of clinically manifest executive dysfunctions, we compared patients with relatively high executive performance to those with a relatively poor performance. To this end, we used individual Executive Function Composite (EFC) scores that can be obtained from the Modified Wisconsin Card Sorting Test (M-WCST; Schretlen, 2010). The M-WCST-based EFC represents a global measure of executive functions which is derived from the number of categories and from the number of perseverative errors on a newly standardized analog of the well-known WCST (Heaton et al., 1993). As this score could be obtained from 20 ALS patients, we established two equally-sized subgroups of ALS patients on the basis of this EFC with a population mean of 100 and a population standard deviation of 15 via a median split. The subgroup that showed below-median performance (ALSef−) showed “borderline” executive dysfunctions on average (*M* = 77.07; *SD* = 13.03; i.e., around 1.53 SDs below the population mean), while the other subgroup (ALSef+) performed at “high average” on the M-WCST (*M* = 114.60; *SD* = 7.07; i.e., around 0.97 SDs above the population mean). Thus, the ALS subgroups differed from each other on the EFC of the M-WCST as expected, but at unforeseen sharpness (by around 2.5 SDs), illustrating the heterogeneity of executive functions in ALS patients. The ALSef− and ALSef+ subgroups did not differ with regard to their mean age (ALSef−: *M* = 63.30, *SD* = 8.26; ALSef+: *M* = 55.20, *SD* = 9.76; *p* = 0.061) or education years (ALSef−: *M* = 13.55, *SD* = 1.67; ALSef+: *M* = 13.65, *SD* = 2.39; *p* = 0.915). The ALSef+ and ALSef− group did not differ with regard to other clinical characteristics, except for the ECAS ALS-specific executive score (ECAS EFS; ALSef−: *M* = 34.10, *SD* = 8.66; ALSef+: *M* = 40.90, *SD* = 3.25; *p* = 0.039), confirming the differences in executive functions revealed by the M-WCST (Table 1). Figure 2 displays histograms of M-WCST EFC and ECAS EFS scores, separately for ALS patients and HC. Inspection reveals that the M-WCST EFC scores sub-divided ALS patients into two, roughly equally sized subgroups (ALSef− and ALSef+, respectively).

### Materials and procedure

Stimulus material was run by Presentation® (Neurobehavioral Systems, Albany, CA). Stimuli were presented for 300 ms against a black background on a computer screen (Eizo EV2416W, Eizo, Hakusan, Japan), subtending a visual angle of 4° × 1° at a viewing distance of 1.45 m. Each stimulus consisted of three white arrows pointing to the left or to the right (Figure 1). Arrows were arranged vertically such that the two outer arrows (“flanker arrows”) either pointed to the same (congruent) or to the opposite (incongruent) direction as the central arrow (“target arrow”). Participants were informed that on each trial, three arrows would appear on the computer screen, and that their task was to focus on the central arrow which would either point to the left or to the right, while ignoring the flanking arrows. They were asked to press the left or right *Ctrl* key on a standard computer keyboard when the central target arrow pointed to the left or right, respectively. Varying the direction of the target arrow (left vs. right) and the congruency of the stimulus array (congruent vs. incongruent) generated four different flanker-target combinations (congruent-left, congruent-right, incongruent-left, incongruent-right). As the direction factor was not of interest for the analyses in this study, congruent-left and congruent-right as well as incongruent-left and incongruent-right stimuli were grouped, and this factor is ignored in the following. Hence, two types of trials are distinguishable: on congruent trials, flanker and target arrows are associated with the same motor response whereas on incongruent trials, flanker and target arrows are associated with contradicting motor responses. Comparing the trial types of the current trial (congruent vs. incongruent) and the type of the respective preceding trial (congruent vs. incongruent) yields four different congruency sequences: preceding trial congruent—current trial congruent (*cC*), preceding trial congruent—current trial incongruent (*cI*), preceding trial incongruent—current trial congruent (*iC*), and preceding trial incongruent—current trial incongruent (*iI*). The response sequence indicates whether the motor response on the current trial had to be repeated (e.g., the target arrow points to the left both on the current trial and the previous trial) or altered (e.g., the target arrow points to the left on the current trial, but had pointed to the right on the previous trial) with respect to the previous trial. Factorial combination of the response sequence with the congruency sequence yields eight experimental conditions (*cC*-repetition, *cC*-alternation, *cI*-repetition, *cI*-alternation, *iC*-repetition, *iC*-alternation, *iI*-repetition, and *iI*-alternation). The distinction between response sequences allows for determining whether the CSE is specific to response repetition trials, as described in the Introduction.

Four hundred and thirty-two (50% congruent, 50% incongruent) stimuli were presented in four blocks in randomized order with a response-stimulus interval of 2000 ms. Response repetition and response alternation trials were equally probable in our task. To acquaint participants to the task, 12 practice trials were presented prior to the experimental phase.

### Electrophysiological recording

Continuous electroencephalogram was recorded with a 32-channel BrainAmp amplifier (Brain Products, Gilching, Germany) and active Ag-AgCl electrodes (Brain Products, Gilching, Germany) mounted on an actiCap (EASYCAP, Herrsching, Germany) according to the international 10–20 system montage. BrainVision Recorder software (Brain Products, Gilching, Germany) was used. Electrode impedance was kept below 10 kΩ. Electrodes were referenced to FCz electrode. To monitor ocular artifacts, vertical (vEOG), and horizontal (hEOG) electrooculogram were recorded with two electrodes positioned at the suborbital ridge and the external ocular canthus of the right eye, respectively.

### Data analysis

#### Behavioral data

RTs and ERs were calculated with respect to the congruency on the current trial and the congruency sequence separately for response repetition and response alternation trials. RTs were obtained by computing the median response latency on correctly completed trials that occurred between 100 and 2000 ms after stimulus onset. ERs were calculated as the proportion of erroneous responses.

#### Electrophysiological data

EEG data were evaluated using BrainVision Analyzer 2.0 (Brain Products, Gilching, Germany). After bandpass filtering (high-pass: 0.5 Hz, 24 dB/oct; low-pass: 70 Hz, 24 dB/oct; notch: 50 Hz), data were screened for artifacts (max. allowed voltage step: 75 μV/ms; lowest allowed activity: 0.5 μV/100 ms), and subjected to an ocular-correction independent component analysis (ICA; Groppe et al., 2008) for further removal of ocular, muscular, and cardiac artifacts. For every possible combination of congruency and response sequence and separately for left and right hand responses, data of correctly completed trials were then segmented into epochs of 1200 ms relative to stimulus onset, baseline corrected (baseline: −200–0 ms), and underwent an artifact rejection procedure [max. allowed voltage difference: 150 μV/200 ms; max. allowed amplitude: -100 μV (min), 100 μV (max)] approved by careful visual inspection in order to exclude remaining artifacts. Data were averaged and re-referenced to a common average reference.

ERP and LRP waves were analyzed for trials that were correctly completed between 100 and 2000 ms after stimulus onset. For the examination of attention-related ERP, we compared posterior negativity amplitudes between ALS patients and HC. As we were primarily interested in differences between ALS patients and HC, we compared ERP waves occurring in the latency area of attention-related posterior negativities between these groups in order to determine potential alterations in attentional processing in ALS patients. Specifically, posterior negativities were calculated as mean amplitudes at O1 and O2 electrodes in the time window from −60 to +60 ms around 196 ms after stimulus onset, where the difference wave of HC—ALS patients reached its maximum at occipital sites. For the analysis of fronto-central N2 waves, peaks were identified in single-subject averages on Cz electrode within a latency area of 200 and 320 ms after stimulus onset (Kopp et al., 1996). The N2 was then calculated as the mean amplitude in the time window from −60 to +60 ms of the individual N2 peak latency. The stimulus-locked LRP (s-LRP) was obtained by subtracting signals recorded at ipsilateral sites (e.g., C3 or C4) from the signals at contralateral sites (e.g., C4 or C3, respectively) separately for the hands, and averaging these difference waves for every subject and condition. s-LRP epochs were created relative to stimulus onset (from −200 ms preceding the stimulus to 1000 ms post-stimulus) separately for all possible combinations of congruency sequence and response sequence for correctly completed trials. Furthermore, the response-locked LRP (LRP-r) was calculated in order to examine the processes before response execution. LRP-r epochs comprised the 1000 ms preceding the motor response as well as the following 350 ms (Eder et al., 2012). LRP onset differences were evaluated in low-pass filtered data (5 Hz, 12 dB/oct) applying the jackknifing method as described in Miller et al. (1998) and Ulrich and Miller (2001; see also Eder et al., (2012) that is a suitable technique for the determination of latency differences (Kiesel et al., 2008). Briefly, grand averages are repeatedly calculated for every condition, every time omitting one participant. By this means, changes in LRP onsets can be determined in each of the resulting grand averages with a higher signal-to-noise ratio than achieved in conventional analyses. We defined the LRP onset as the point in time where the LRP amplitude first reached 50% of its maximum amplitude (i.e., the maximum amplitude in that condition including all participants), as proposed by Miller et al. (1998; see also Eder et al., 2012). The resulting values are then subjected to an ANOVA. As this method further underestimates the actual between-subjects variance, *F*-values need to be corrected so that $F_{c}=F∕{\left(n-1\right)}^{2}$ (Miller et al., 1998; Ulrich and Miller, 2001). *F*_*c*_ is then compared to the critical *F*-values. As EEG data obtained from 21 ALS patients and 20 HC were analyzed, but equal sample sizes are required for the comparison of jackknifed data described here, we excluded the patient with the noisiest EEG from the LRP onset analyses (Ulrich and Miller, 2001). LRP amplitudes were measured as individual peak minima at the C3-C4 electrode pair after 5-Hz (12 dB/oct) low-pass filtering.

For statistical analyses, repeated measurement ANOVA was used with group (ALS vs. HC) as between-subject factor and conditions (congruency on *current* trial: congruent vs. incongruent; congruency on *previous* trial: congruent vs. incongruent; response sequence: repetition vs. alternation) as within-subject factors. For the analyses of posterior negativity amplitudes, the factor electrode site (O1 vs. O2) was added and the factors congruency on previous trial and response sequence were not included as these effects were not of interest for the present study. Effect sizes for ANOVA results were calculated as $\eta_{p}^{2}$. Greenhouse-Geisser corrections were used where appropriate. For *post-hoc* tests, *p-*values are reported after Bonferroni correction for multiple comparisons. For correlation analyses, Spearman-Brown correlation coefficients were calculated. To determine potential associations between executive performance, task performance and electrophysiological measures, we correlated the EFC of the M-WCST and the ECAS EFS with RT, ER and the amplitudes of the posterior negativity, N2, s-LRP and LRP-r, each averaged across all conditions of current congruency, previous congruency and response sequence. The posterior negativity amplitudes were averaged over O1 and O2 electrodes for the correlation analyses.

## Results

### Neuropsychological tests

The results obtained from neuropsychological testing are displayed in Table 1. ALS patients did not show statistically significant impairments in performance in comparison to HC in any of the tests that were applied.

This study brought into focus whether flanker congruency (i.e., CE) and/or congruency sequence (i.e., CSE) exerted differential effects on behavioral, ERP and LRP responses in ALS patients and HC. The results thus primarily report the effects of group, of flanker congruency (i.e., CE), of the congruency sequence by response sequence interaction (i.e., CSE by Response Sequence), as well as the interactions between group and these two repeated-measures manipulations.

### Behavioral data

#### Response times

**Figure 3:**
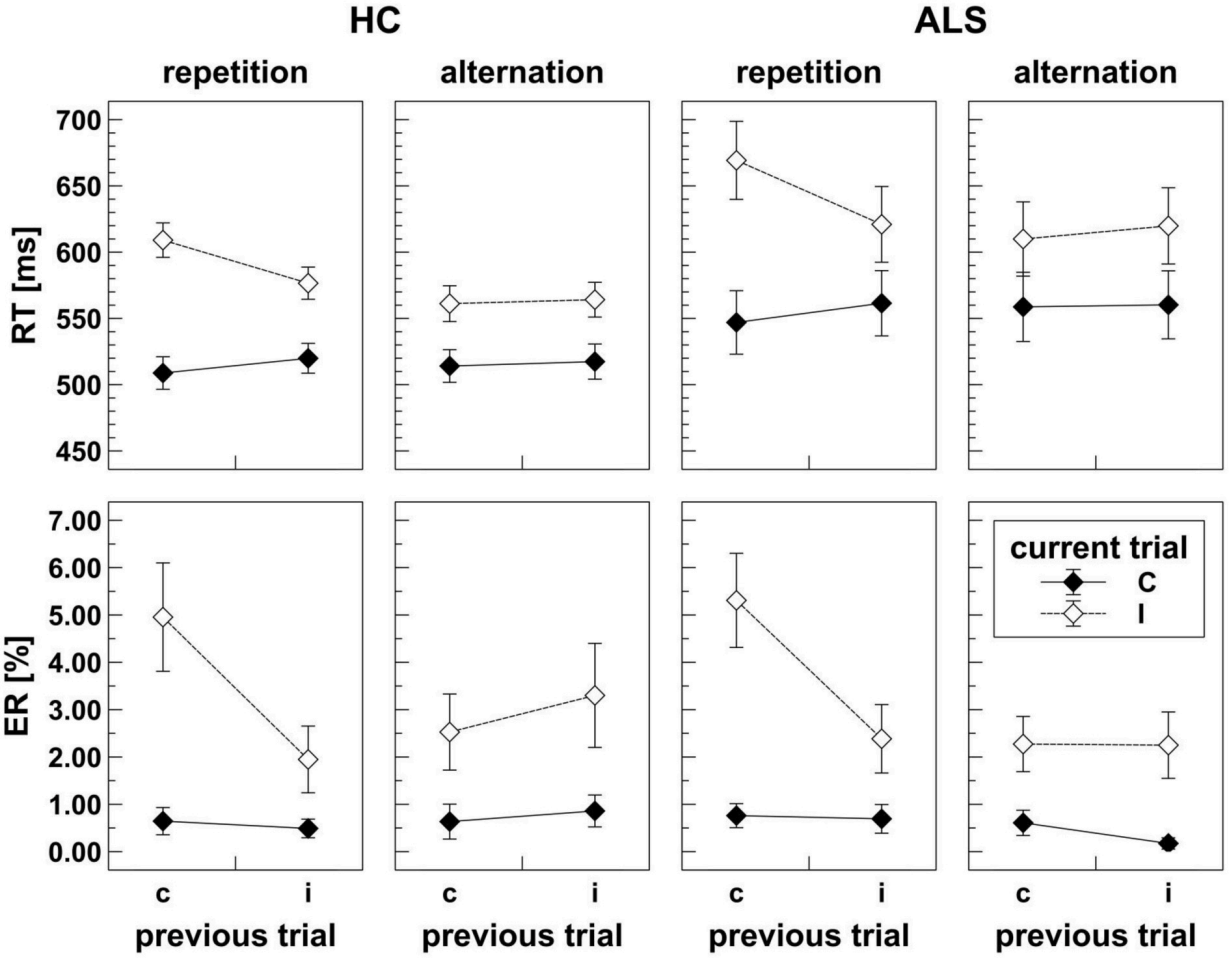
**Behavioral data obtained in the flanker task for HC (left panels) and ALS patients (right panels)**. Upper panels display response times (RT) separately for response repetition and response alternation trials. Lower panels display error rates (ER) separately for response repetition and response alternation trials. Lower case *c* (congruent) and *i* (incongruent) on the abscissa indicate previous trial congruency level; upper case *C* (congruent) and *I* (incongruent) on separate lines indicate current trial congruency level. For HC as well as for ALS patients, response times and error rates are modulated by the typical effects of congruency and a congruency sequence effect that is present in response repetition, but absent in response alternation trials.

As shown in Figure 3, ALS patients (mean RT = 593 ms) responded slower than HC (mean RT = 546 ms); however, this apparent response slowing did not reach statistical significance, *F*_(1, 39)_ = 2.60, *p* = 0.115, $\eta_{p}^{2}=0.063$. There was a significant CE, *F*_(1, 39)_ = 192.19, *p* < 0.001, $\eta_{p}^{2}=0.831$, and a significant CSE by Response Sequence interaction, *F*_(1, 39)_ = 56.27, *p* < 0.001, $\eta_{p}^{2}=0.591$, indicating that these independent manipulations exerted massive effects on RT variation. As expected, congruent trials showed faster mean RT (536 ms) than incongruent trials (604 ms). On response repetition trials (cf. Figure 1), mean RT on *cC* and *iI* trials amounted to 528 ms and 599 ms, respectively, which is shorter than those on *iC* and *cI* trials (541 ms and 639 ms), compatible with the expected CSE on these trials. On response alternation trials (cf. Figure 1), mean RT on *cC* and *iI* trials amounted to 536 and 592 ms, respectively, which is roughly identical to those on *iC* and *cI* trials (539 and 586 ms), compatible with the expected absence of the CSE on these trials. Group did not significantly modulate the CE, *F*_(1, 39)_ = 1.14, *p* = 0.292, $\eta_{p}^{2}=0.028$, nor the CSE by Response Sequence interaction, *F*_(1, 39)_ = 3.34, *p* = 0.075, $\eta_{p}^{2}=0.079$.

#### Error rates

Visual inspection revealed that error data were not normally distributed. ANOVA results did not change when we repeated the analyses with arcsine-transformed or log-transformed (*y* = log (1.1 − *x*)) data. For simplicity, we report the results of untransformed ER data here. ER were similar for ALS patients (mean ER = 1.8%) and HC (mean ER = 1.9%), such that the main effect of Group did not reach statistical significance, *F*_(1, 39)_ = 0.04, *p* = 0.846, $\eta_{p}^{2}=$ 0.001. There was a significant CE, *F*_(1, 39)_ = 40.98, *p* < 0.001, $\eta_{p}^{2}=$ 0.512, and a significant CSE by Response Sequence interaction, *F*_(1, 39)_ = 17.75, *p* < 0.001, $\eta_{p}^{2}=$ 0.313, indicating that these manipulations exerted strong effects on ER variation. As expected, fewer errors were committed on congruent trials (0.6%) than on incongruent trials (3.1%). On response repetition trials (cf. Figure 1), ER on *cC* and *iI* trials amounted to 0.7 and 2.2%, respectively, whereas ER on *iC* and *cI* trials were 0.6 and 5.1%, compatible with the expected CSE on these trials. On response alternation trials (cf. Figure 1), ER on *cC* and *iI* trials amounted to 0.6 and 2.8%, respectively, which is roughly identical to those on *iC* and *cI* trials (0.5 and 2.4%), compatible with the expected absence of the CSE on these trials. Group did not significantly modulate CE nor the CSE by Response Sequence interaction (all *F* < 0.01, all *p* >0.05).

### Electrophysiological data

#### Posterior negativity

**Figure 4:**
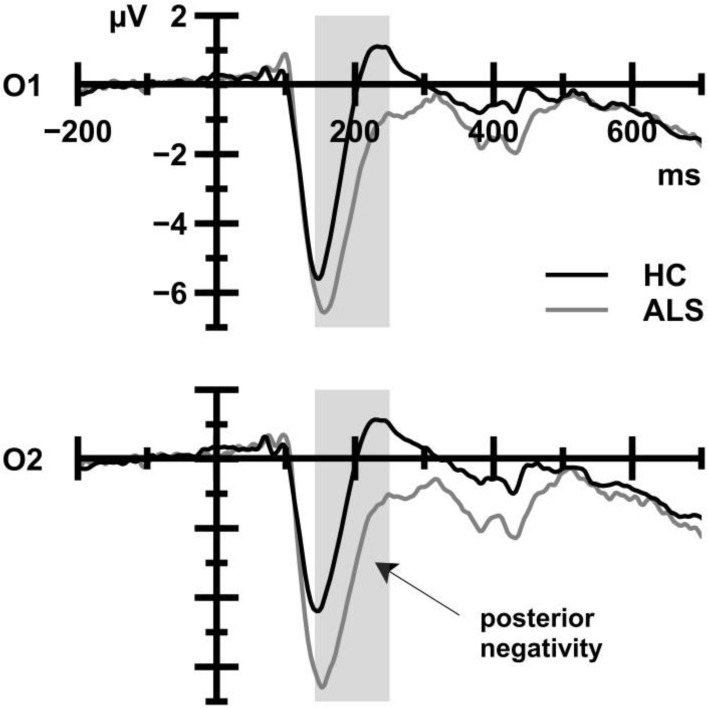
**ERP waveforms that were obtained from HC (black) and ALS patients (gray) averaged over all trial types**. The shaded area indicates the time window for analyses. ALS patients show more pronounced posterior negativities at occipital sites compared to HC.

Figure 4 displays posterior negativities that were recorded from ALS patients and HC. ALS patients (−3.69 μV) showed enhanced amplitudes compared to HC (−1.43 μV), as reflected in a statistically significant main effect of Group, *F*_(1, 39)_ = 6.98, *p* = 0.012, $\eta_{p}^{2}=$ 0.152. There was no main effect of CE, *F*_(1, 39)_ = 0.08, *p* = 0.782, $\eta_{p}^{2}=$ 0.002, and the Group by CE interaction failed to reach statistical significance, *F*_(1, 39)_ = 3.25, *p* = 0.079, $\eta_{p}^{2}=$ 0.077.

#### N2

**Figure 5:**
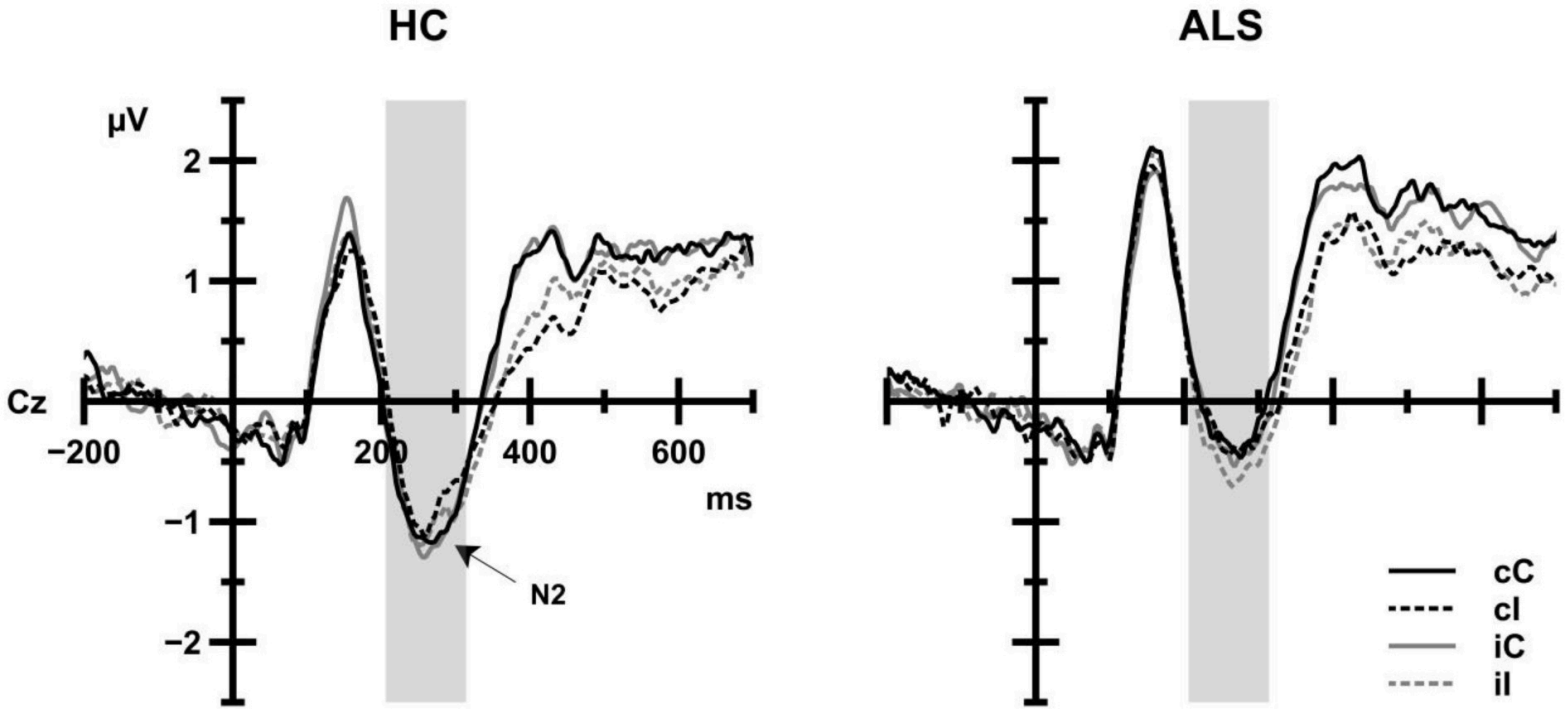
**Grand average ERP activity of HC (left panel) and ALS patients (right panel) as a function of current trial congruency (solid lines: congruent trials, dashed lines: incongruent trials) at Cz electrode**. Black lines indicate that the previous trial was congruent; gray lines indicate that the previous trial was incongruent. The shaded area indicates the time window for analyses. No group differences were observed.

As shown in Figure 5, N2 amplitudes in ALS patients (−0.53 μV) and in HC (−0.92 μV) achieved similar levels. Group, CE, and the CSE by Response Sequence interaction as well as interactions between Group and CE or CSE by Response Sequence were not statistically significant (all *F* < 1.84 all *p*>0.05). However, N2 amplitude was modulated by the congruency of the trial *preceding* the current trial, *F*_(1, 39)_ = 5.19, *p* = 0.028, $\eta_{p}^{2}=$ 0.117, such that N2 amplitudes were more negative when the preceding trial was incongruent (−0.79 μV) than when the preceding trial was congruent (−0.67 μV).

#### s-LRP onset latencies

**Figure 6:**
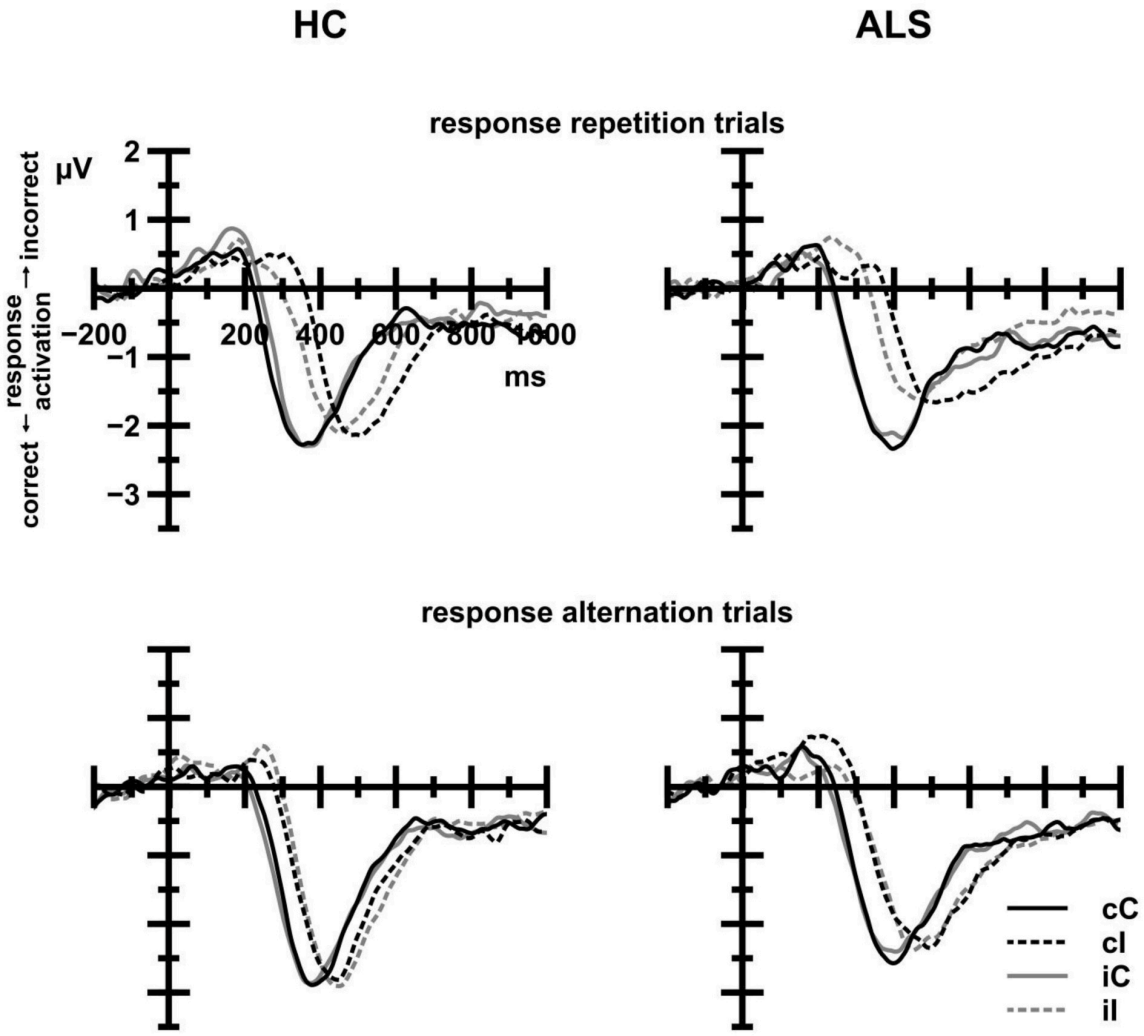
**Stimulus-locked LRP waveforms for HC (left panels) and ALS patients (right panels) as a function of current trial congruency (solid lines: congruent trials, dashed lines: incongruent trials)**. Black lines indicate that the previous trial was congruent; gray lines indicate that the previous trial was incongruent. Positive deflections reflect incorrect response activation; negative deflections reflect correct response activation. Response repetition trials (upper panels) exhibit a previous congruency by current congruency interaction that is absent in response alternation trials (lower panels). There were no differences between ALS patients and HC.

Inspection of Figure 6 reveals that s-LRP onset latencies were strongly modulated by CE, *F*_*c*(1, 38)_ = 53.51, *p* < 0.001, $\eta_{p}^{2}=$ 0.585, with shorter latencies on congruent (287 ms) compared to incongruent trials (370 ms). The overall effect of CE was further modulated by Response Sequence, *F*_*c*(1, 38)_ = 5.99, *p* = 0.019, $\eta_{p}^{2}=$ 0.136, indicating that onset latencies on congruent trials were shorter on response repetition (282 ms) than on response alternation trials (291 ms), whereas onset latencies on incongruent trials were shorter on response alternation (351 ms) than on response repetition trials (388 ms). Group, alone or in interaction with CE or CSE by Response Sequence, did not reach statistical significance (all *F* < 0.23, all *p* > 0.05).

#### LRP amplitudes

##### s-LRP

**Figure 7:**
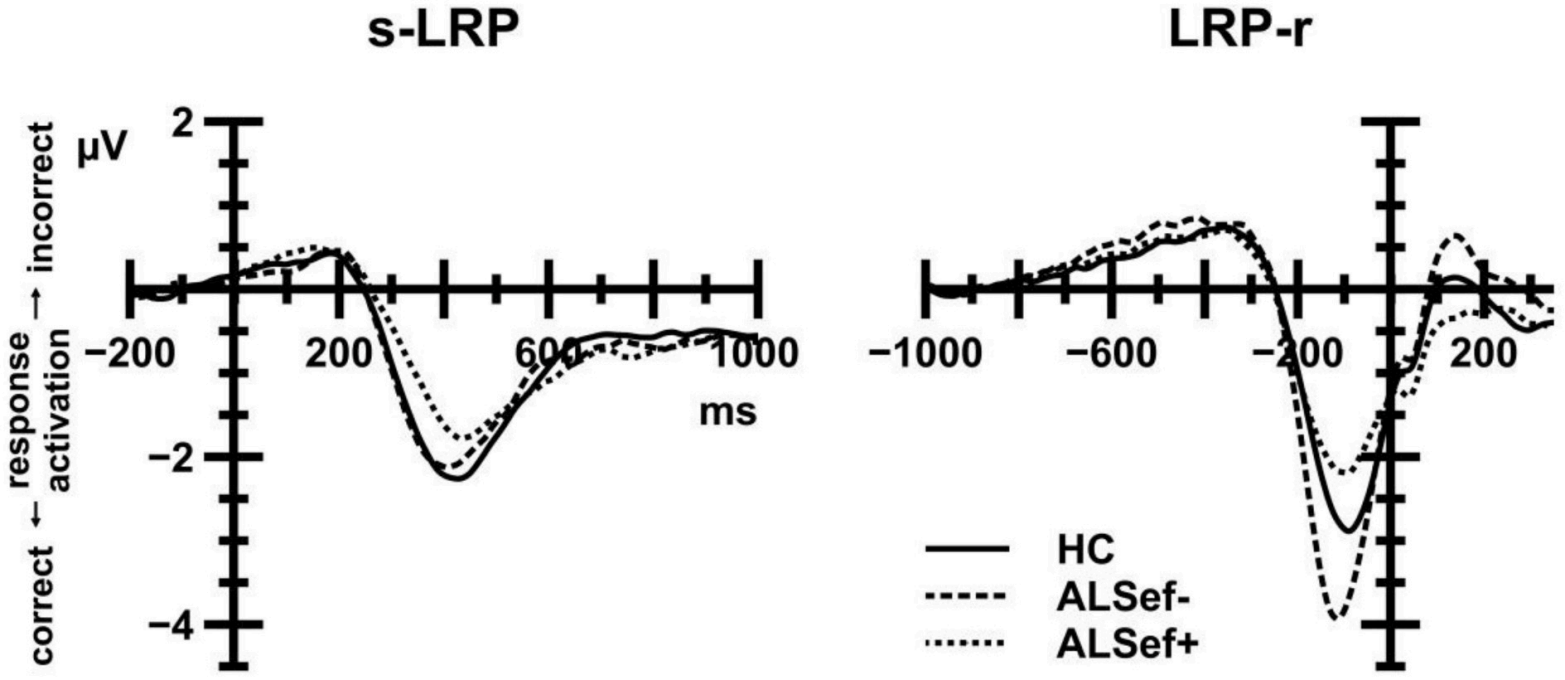
**Stimulus-locked (left panel) and response-locked (right panel) LRP waveforms**. Positive deflections reflect incorrect response activation; negative deflections reflect correct response activation. To illustrate the relation between M-WCST performance and LRP amplitudes, ALS patient data were subdivided: dashed lines show LRP data for ALS patients with below-median performance on the M-WCST (ALSef−); dotted lines show LRP data for ALS patients with above-median performance on the M-WCST (ALSef+). These patient subgroups differed markedly with regard to their LRP-r amplitude preceding the overt response.

Figure 7 (left panel) displays s-LRP amplitudes that were recorded from ALS patients and HC. In short, s-LRP amplitudes were unaffected by Group, either alone or in interaction with CE and the CSE by Response Sequence interaction (all *F* < 1.80, all *p*>0.05). However, s-LRP amplitudes were enhanced on response alternation (−3.43 μV) compared to response repetition trials (−2.86 μV), giving rise to a statistically significant Response Sequence effect, *F*_(1, 39)_ = 32.07, *p* < 0.001, $\eta_{p}^{2}=$ 0.451.

##### LRP-r

Figure 7 (right panel) displays LRP-r amplitudes that were recorded from ALS patients and HC. In short, LRP-r amplitudes were unaffected by Group, either alone or in interaction with CE and the CSE by Response Sequence interaction (all *F* < 1.10, all *p*>0.05). LRP-r amplitudes were enhanced on response alternation (−3.46 μV) compared to response repetition trials (−3.20 μV), giving rise to a statistically significant Response Sequence effect, *F*_(1, 39)_ = 11.47, *p* = 0.002, $\eta_{p}^{2}=$ 0.227.

### Relationships with executive functions

#### ALS subgroup comparisons

Comparisons between HC, ALSef+, and ALSef− patients did not reveal statistically significant effects of Group on RT, ER, N2 amplitudes and s-LRP amplitudes (all *F* < 2.74, all *p*>0.05). Note that we did not compare s-LRP onset latencies because equal sample sizes are required for jackknifing (Ulrich and Miller, 2001), and the HC group comprised 20 individuals, whereas the ALSef+ and ALSef− patient groups each comprised 10 individuals.

With regard to posterior negativity amplitudes (see Figure 4), the comparison between HC, ALSef+, and ALSef− patients yielded a statistically significant Group effect, *F*_(2, 37)_ = 4.59, *p* = 0.017, $\eta_{p}^{2}=$ 0.199. *Post-hoc* tests revealed that posterior negativity amplitudes were enhanced in the ALSef− group (−4.44 μV) in comparison to HC (−1.43 μV), *p* = 0.020, while the remaining group comparisons revealed statistically non-significant results [HC vs. ALSef+ (−3.36 μV): *p* = 0.216, ALSef− vs. ALSef+: *p*>0.999].

With regard to LRP-r amplitudes (see Figure 7, right panel), the comparison between HC, ALSef+, and ALSef− patients yielded a statistically significant Group effect, *F*_(2, 37)_ = 3.54, *p* = 0.039, $\eta_{p}^{2}=$ 0.160. *Post-hoc* tests revealed that LRP-r amplitudes were enhanced in ALSef− (−4.37 μV) in comparison to ALSef+ patients (−2.61 μV), *p* = 0.040, while the remaining group comparisons revealed statistically non-significant results [HC (−3.22μV) vs. ALSef−: *p* = 0.173; HC vs. ALSef+: *p* = 0.915].

#### Correlation analyses

Since the sample size of the ALS subgroup comparisons was rather small, correlation analyses were performed in order to substantiate the reliability of the effects. Table 2 shows the results of these correlation analyses, i.e., Spearman rank correlation coefficients, separately for ALS patients (above the diagonal) and HC (below the diagonal). We chose Spearman correlations rather than Pearson correlations because they are less sensitive to strong outliers. For sake of brevity, we only comment on significant correlations between indicators of executive dysfunctions and behavioral and electrophysiological measures that were obtained on the flanker task. To begin with, ECAS EFS correlated negatively with ER (*r*_*s*_ = −0.446, *p* = 0.043) in ALS patients, indicating that better performance on the executive part of the ECAS was associated with more accurate performance. Further, LRP amplitudes (s-LRP, *r*_*s*_ = 0.564, *p* = 0.010; LRP-r, *r*_*s*_ = 0.574, *p* = 0.008) correlated with the M-WCST EFC in ALS patients, but not in HC (all *p*>0.05). It is worthy of note that these two correlation coefficients differed between ALS patients and HC (s-LRP, *z* = 1.98, *p* = 0.048; LRP-r, *z* = 1.95, *p* = 0.051).

**Table 2:** **Correlations of executive performance in neuropsychological assessments, and behavioral performance and electrophysiological measures obtained on the flanker task for ALS patients (above diagonal, shaded) and HC (below diagonal)**.

**ALS (above diagonal)/HC (below diagonal)**	**M-WCST EFC**	**ECAS EFS**	**RT**	**ER**	**Posterior negativity amplitude**	**N2 amplitude**	**s-LRP amplitude**	**LRP-r amplitude**
M-WCST EFC	–	0.384	0.360	−0.230	0.195	−0.003	0.564^**^	0.574^**^
ECAS EFS	0.140	–	0.203	−0.446^*^	0.319	−0.319	0.169	0.199
RT	−0.384	0.005	–	−0.194	0.066	0.009	0.573^**^	0.514^*^
ER	0.079	−0.118	−0.311	–	−0.499^*^	−0.095	−0.231	−0.330
Posterior negativity amplitude	0.191	−0.395	−0.044	−0.122	–	−0.049	0.364	0.496^*^
N2 amplitude	0.348	0.160	0.162	−0.374	−0.233	–	0.174	0.169
s-LRP amplitude	−0.040	−0.121	0.377	−0.358	0.122	0.477^*^	–	0.956^**^
LRP-r amplitude	−0.017	−0.104	0.359	−0.418	0.111	0.499^*^	0.971^**^	–

* *p < 0.05*,

** *p < 0.01*.

## Discussion

We examined 21 ALS patients and 20 age-, gender-, and education-matched control participants with a battery of neuropsychological tests and a non-verbal version of the flanker task. ALS patients showed normal performance in their response times (RT), suggesting that conflict processing and its contextual modulation are unaffected by the disease. This conclusion that can be drawn from the behavioral data was further corroborated by the ERP and LRP data in that no evidence for altered neural indices of conflict processing and its contextual modulation could be discerned. More specifically, neither N2 amplitudes nor s-LRP onset latencies were altered in ALS patients compared to HC. However, we made three more subtle, nonetheless potentially important, observations. First, ALS patients showed enhanced posterior negativity amplitudes at occipital electrodes, and this ERP enhancement was somewhat more pronounced in those patients who showed clinical evidence for some degree of executive dysfunctions (EDF) as assessed by the M-WCST EFC. Second, subgroup analyses and correlation analyses converged in the finding that the same subgroup of ALS patients showed enhanced LRP amplitudes in comparison to ALS patients without clinical signs of EDF. The LRP amplitude data suggest that the presence of EDF in ALS might be associated with functional alterations in motor regions of the cerebral cortex. Third, the presence of EDF in ALS as assessed by the ECAS EFS was associated with more error-prone behavior (ER) on the flanker task, putatively mediated through functional alterations in prefrontal regions of the cerebral cortex (Luks et al., 2010).

### Theoretical integration of the findings

**Figure 8:**
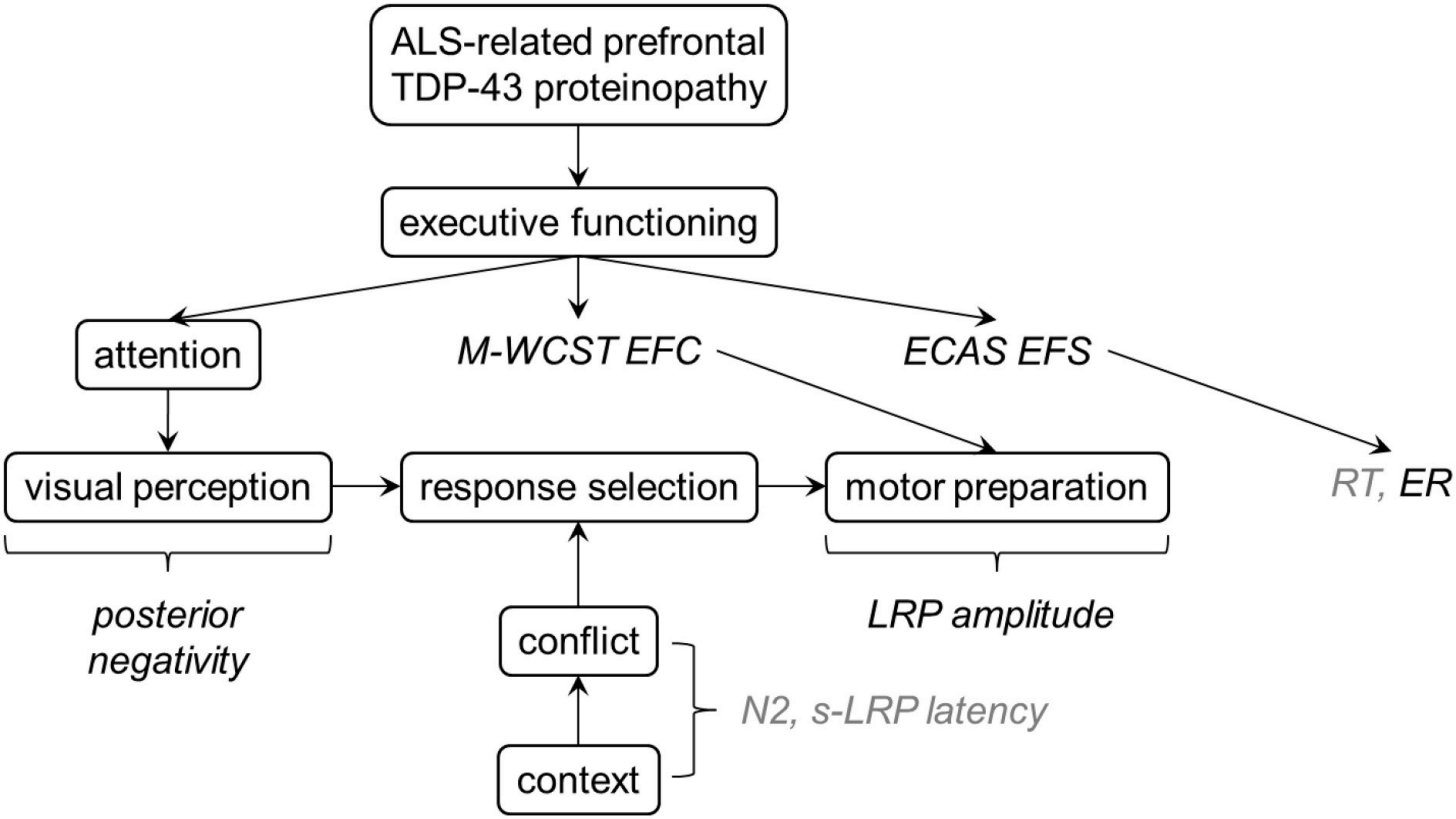
**A suggestion for post-hoc theoretical interpretation of the data**. ALS-related prefrontal TDP-43 proteinopathy should be associated with measurable interindividual differences in executive functions. While response selection seems to be relatively unaffected by ALS (as revealed by normal RT, N2 amplitudes and s-LRP), enhanced posterior negativities in ALS patients might reflect functional compensation of executive deficits by stronger modulation of visual processing. LRP amplitudes were associated with EDF in ALS patients, pointing to a potential link between functional dysregulation in prefrontal and motor areas of the cortex. More error-prone behavior on the flanker task was also related to EDF in ALS patients.

Figure 8 presents a post-hoc synthesis of these data. Note that due to the exploratory character of our study, this synthesis can only represent a preliminary interpretation of the results discussed here. Further studies are required to confirm or disconfirm these findings, and to finally understand the nature of altered information processing in ALS and the cortical correlates thereof. Here, we consider the ALS-related prefrontal TDP-43 proteinopathy as the final (i.e., neurobiological) common pathway (Arai et al., 2006; Neumann et al., 2006; Geser et al., 2008). We assume that individual differences in ALS-related prefrontal proteinopathy are associated with individual differences in clinically manifest EDF, as assessed by the M-WCST EFC and the ECAS EFS. Our non-verbal flanker study was conducted to analyze how this prefrontal proteinopathy affects information processing from perception to action, particularly conflict processing and its contextual modulation. As can be seen, behavioral measures (RT, ER) do not allow decomposing information processing into its constituent parts because altered perception, response selection and motor preparation might induce, alone or in dynamic interplay, alterations in RT or ER. The desired decomposition can, however, be achieved by measuring ERP and LRP because they provide relatively specific measures of neural substrates of these constituent parts. Specifically, posterior negativities reflect attentional modulation of (visual) perception, N2 amplitudes and s-LRP onset latencies are sensitive to conflict processing and its contextual modulation, and LRP amplitudes offer insights into cortical mechanisms of motor preparation. Our findings suggest four major conclusions: First, response selection seems generally unaffected by the disease. In particular, conflict processing and its contextual modulation are spared by the ALS-related prefrontal proteinopathy, as revealed by the unaltered response times (RT), N2 amplitudes and s-LRP onset latencies in ALS patients (Figure 8, italics in light gray). Second, declines in executive abilities appear to be functionally compensated by increased modulation of visual processing by frontoparietal networks in ALS, as revealed by enhanced posterior negativities (Figure 8, italics in black). Third, individual differences in clinically manifest EDF, as assessed by the M-WCST EFC, are associated with enhanced LRP amplitudes in ALS patients, pointing to a potential link between functional dysregulation in prefrontal and motor areas of the cerebral cortex in ALS. Fourth, individual differences in clinically manifest EDF, as assessed by the ECAS EFS, are associated with error-prone behavior (ER) on the flanker task, pointing to prefrontal functional dysregulation (Luks et al., 2010). For sake of brevity, we will only shortly comment on each of these conclusions in the remainder of this discussion.

### Conflict processing in ALS patients

The ability to efficiently select motor responses appears to be unaffected by ALS, both under non-conflicting as well as under conflicting conditions. Further, we could not discern evidence (behavioral, neural) for ALS-related alterations with regard to the well-documented (see Section Introduction) contextual modulation of conflict processing. Of course, null findings should be treated with caution; however, they stand in contrast to earlier findings from our group that we obtained from PD patients (Rustamov et al., 2013). In this flanker study, we found that PD is associated with alterations in the contextual modulation of conflict processing. Further research is required to examine directly potential dissociations between ALS and PD with regard to the contextual modulation of conflict processing.

### Attentional modulation of visual perception in ALS patients

As outlined in the Introduction, posterior negativities are subject to prefrontal modulation (Barceló et al., 2000). Following the evidence from that study, modulations of posterior negativities have their origin on frontoparietal attention networks (Ptak, 2012; Vossel et al., 2014). According to this approach, attentional control involves the setting of task-driven priorities to bias competition in visuospatial feature maps thus affecting the information gain from task-relevant and task-irrelevant parts of a visual scene. Attentional control as it applies to flanker tasks is discussed in detail in Rustamov et al. (2014).

ALS patients, in particular those with clinically detectable EDF, showed enhanced posterior negativity amplitudes in comparison to HC. Given evidence for prefrontal control over posterior negativity amplitudes, these data might indicate a task-driven “sharpening” of visual processing (Rustamov et al., 2014), putatively in the service of functional compensation (Reuter-Lorenz and Cappell, 2008) and probably through additional mechanisms recruited in frontoparietal attention networks (Ptak, 2012; Vossel et al., 2014). If so, the present posterior negativity finding is in line with multiple neuroimaging studies that used various paradigms, and that demonstrated a diversity of patterns of enhanced cortical activity in ALS patients (Kew et al., 1993b; Schoenfeld et al., 2005; Han and Ma, 2006; Stanton et al., 2006; Lulé et al., 2007; Dounaud et al., 2011; Goldstein et al., 2011; Mohammadi et al., 2011, 2015; Cosottini et al., 2012; Witiuk et al., 2014; see Turner et al., 2012, for review).

### Motor preparation in ALS patients

The LRP is a quantification of the inequality of the ERPs contralateral and ipsilateral to the hand making the response. The two competing hands, left vs. right, are in equal activation until one side gets the advantage. When the disparity between the two hands becomes non-zero, the LRP has its onset. LRP amplitudes are of considerable interest in the light of a number of studies that showed that LRP amplitudes are enhanced in elderly participants in comparison to young participants (Yordanova et al., 2004; Roggeveen et al., 2007; Wild-Wall et al., 2008; Vallesi and Stuss, 2010; Cespón et al., 2013; Cid-Fernández et al., 2014). According to one hypothesis, increased LRP amplitudes in older adults arise from reduced inhibition (mediated by GABAergic synapses) within the motor cortex (Roggeveen et al., 2007). When inhibition is weaker, both activations ultimately build up to higher levels, and enhanced LRP amplitudes will result to the extent of stronger disinhibition for the responding hand than for the competing hand. Alternatively, enhanced LRP amplitudes were associated with dysregulation in high-level control networks on the basis of the compensation hypothesis (Reuter-Lorenz and Cappell, 2008), suggesting that enhanced LRP amplitudes may be related to additional mechanisms recruited for maintaining motor performance (Wild-Wall et al., 2008).

In contrast to normal aging, ALS itself does not seem to be associated with enhanced LRP amplitudes. However, subgroup analyses and correlation analyses showed that clinically detectable ALS-related EDF (as assessed by the M-WCST EFC) are related to enhanced LRP amplitudes. The specificity of the relationship between EDF and enhanced LRP amplitudes suggests that the LRP amplitude enhancement in ALS patients with EDF might occur as a corollary of the dysregulation in prefrontal control networks that in turn may be related to additional mechanisms recruited for maintaining motor performance.

### Error-proneness in ALS patients

The ECAS (Abrahams et al., 2014) allowed examining aspects of executive functions beyond those assessed by the M-WCST. Performance deficits on the ECAS EFS predicted higher ER on the flanker task. Luks et al. (2010) found that atrophy of the left hemisphere dlPFC and ACC in 65 patients with various neurodegenerative diseases (frontotemporal lobar degeneration, Alzheimer's disease, corticobasal degeneration, or progressive supranuclear palsy, but not ALS) predicted higher ER on the flanker task. It is thus possible that the relationship between ECAS EFS and ER might be mediated by dysfunction of the left hemisphere dlPFC and ACC. The proposed relationship remains to be elucidated in future studies using structural and functional imaging techniques.

### Study limitations

Replication in independent, larger samples of ALS patients is warranted, in particular to ensure the validity of the subgroup comparisons. Future studies should also consider the multidimensional nature of executive functions (Miyake et al., 2000). Finally, we suggest comparing ALS patients with patients suffering from other neurodegenerative diseases in future studies. The accomplishment of a comparative approach would allow investigating the degree of specificity of cognitive and behavioral disturbances that are associated with ALS, ultimately leading to a better understanding of the neural underpinnings of these psychological disturbances.

## Conclusions

This article demonstrates the utility of ERP and LRP measures that provide the means for decomposing psychological disturbances associated with ALS into constituent parts (Figure 8). Here, we focused on executive functioning which is important for the ability to predict behavioral sequelae and course of the disease (Olney et al., 2005; Chiò et al., 2010; Elamin et al., 2011; Lillo et al., 2012; Montuschi et al., 2015). Our results exemplify how electrophysiological measures might contribute to a better clinical assessment as well as to a more rigorous scientific investigation of cognitive dysfunctions in ALS patients (see also Raggi et al., 2010; Lange et al., 2015).

Accumulating evidence about the heterogeneous nature of ALS raised the awareness about its presumably multifaceted etiology and phenomenology. Recently, this has led to the hypothesis of ALS being a general term for a variety of related yet distinct disorders, rather than a clearly defined disease (Goldstein and Abrahams, 2013; Turner et al., 2013). Our results thus provide support for the need of studies disentangling these ALS subtypes in order to adequately characterize the individual problems associated with the disease and to find the optimal treatment for individual patients. Our data highlight the promising role of the ERP technique as a tool to overcome difficulties associated with purely behavioral examination techniques (Goldstein and Abrahams, 2013), and they support the previously suggested distinction of ALS with and without cognitive involvement (Ringholz et al., 2005; Lillo and Hodges, 2010; Phukan et al., 2012).

## Author contributions

Conceived the study: RD, SP, BK. Designed the experiment: CS, SF, SP, BK. Performed the experiment: CS, SF, MV, SA. Analyzed the data: CS, SF, MV, FL, SA, BK. Wrote the manuscript: CS, SF, MV, FL, RD, SP, BK. All authors provided critical revisions to the manuscript and approved the final version of the paper.

### Conflict of interest statement

The authors declare that the research was conducted in the absence of any commercial or financial relationships that could be construed as a potential conflict of interest.
